# Enhanced Oxidation of *p*-Toluidine Using Supported Zeolite Nanoparticles

**DOI:** 10.3390/molecules28155737

**Published:** 2023-07-29

**Authors:** Khadijah H. Alharbi, Walaa Alharbi, Sultan Alhayyani, L. Selva Roselin, Rosilda Selvin

**Affiliations:** 1 Department of Chemistry, Science and Arts College, King Abdulaziz University, Rabigh 21911, Saudi Arabia; wnhalharbe@kau.edu.sa (W.A.); salhayyani@kau.edu.sa (S.A.); slous@kau.edu.sa (L.S.R.); 2 Department of Basic Sciences and Humanities, Don Bosco Institute of Technology, Kurla (W), Mumbai 400 070, India; selvinrosilda@yahoo.com

**Keywords:** oxidation of *p*-toluidine, magnetite, titanium silicalite-1, 4,4′-dimethylazobenzene, 4,4′-dimethylazoxybenzene

## Abstract

Supported nanomaterials are becoming increasingly important in many industrial processes because of the need to improve both the efficiency and environmental acceptability of industrial processes. The unique properties of supported nanomaterials have attracted researchers to develop efficient catalytic materials in nanoscale. The extremely small size of the particles maximizes the surface area exposed to the reactant, allowing more reactions to occur. The environmental hazards resulting from the conventional manufacturing procedures for organic fine chemicals and intermediates by classical oxidation catalysis using mineral acids have forced chemical industries to seek less polluting processes. The present study aimed to oxidize *p*-toluidine by hydrogen peroxide in the presence of magnetite supported on nanocrystalline titanium silicalite-1 (M/NTS) zeolite at ambient temperature. The products detected are 4,4′-dimethylazobenzene as major product and 4,4′-dimethylazoxybenzene as minor product. Good selectivity, low cost, low wastage of materials and enhanced environmental friendliness of heterogeneous magnetite nanoparticle supported zeolite catalysts were observed. The effect of various reaction parameters such as mole ratio, catalyst weight and reusability of catalyst were studied. At the optimum reaction conditions, the oxidation activity of M/NTS catalyst was compared with M/NS catalyst, and it was found that titanium in the framework of M/NTS provided higher activity and selectivity.

## 1. Introduction

The prime focus of today’s chemists is on the production of industrially important organic compounds through economically viable and environment-friendly catalytic routes [1,2,3]. Several catalyst systems have been developed in the past. The discovery and continuing development of new and innovative catalysts has stimulated studies on different supports [4,5,6].

Toluidines are methyl-substituted aromatic amines, used in the manufacture of various organic chemicals. The *p*-isomer has antimicrobial activity and is used for wastewater treatment. Also, it is used as a reagent to analyze lignin and nitrile phloroglucinol [7]. Oxidation of *p*-toluidine produces a number of products depending on the oxidation method (electrochemical/thermal), nature of the oxidants and experimental conditions used. These products are considered as industrially valued products for the manufacture of dyes and other organic chemicals.

When a mixture of aniline, *p*-toluidine and o-toluidine were oxidized in a sulfuric acid medium with a platinum electrode, rosaniline was formed [8]. The electrochemical oxidation method has its own drawbacks of high cost of the electrodes, the risk of formation of unwanted by-products and the energy demand.

The oxidation of *p*-toluidine using oxidizing agents such as KMnO_4_, K_2_Cr_2_O_7_, sulfuric acid and K_3_Fe(CN)_6_ was reported, and these catalysts resulted in the formation of a trimer and/or tetramer of *p*-toluidine [9,10,11]. Biocatalytic oxidation using enzyme peroxidase leads to the formation of both a trimer and a tetramer along with other by-products [12]. The selectivity towards trimer and tetramer depends on the oxidant type, pH of the medium, temperature and concentration of *p*-toluidine [13]. Ritu Singh investigated the oxidation of *p*-toluidine by sodium dichromate and analyzed it iodometrically and spectrometrically (at 530 nm) in an aqueous acidic medium. The kinetic behavior of the reaction was altered by *p*-toluidine and hydrogen ion concentration. This is because of the formation of an intermediate complex that breaks down into two different reaction paths [14]. The oxidation of o-toluidine and *m*-toluidine with lead acetate produced better yield of azo compound in comparison to *p*-toluidine [15]. Oxidation of o-toluidine with periodate oxidant in an acetone–water medium illustrates that the reaction was one of first-order kinetics with respect to both oxidant and substrate. Further, it was found that the reaction was pH-dependent, and the reaction slowed down while decreasing the dielectric constant and increased while increasing the ionic strength [16]. These catalysts cause severe environmental pollution, as they require rigorous control of the experimental conditions and leaching of transition metal ions [17]. In addition, the product selectivity was low, as it involved various multistage reaction process [18].

Owing to the disadvantages of homogenous catalysts, they are replaced by numerous heterogeneous catalysts such as metals, metal oxides, supported metal complexes, metal catalysts supported on mesoporous materials, metallosilicalites, hydrotalcites and heteropoly compounds. These are supposed to be promising catalysts because of the advantage of great catalyst reusability and shape selectivity [19,20,21]. Among the various solid catalysts, zeolites are extensively studied. Zeolites are crystalline aluminosilicates having regular arrangements of micropores with high surface area, exchangeable cations and shape selectivity [22,23,24,25,26,27]. The shape selectivity of catalysts is used for various catalytic reactions over zeolite heteroatom-based sites such as acidic and basic sites and Ti and Fe sites [28]. TS-1 molecular sieve possesses nanopores and so exhibits higher activity [29], excellent adsorption properties [30] and the unique shape-selective catalytic function of MFI topology [31]. Titanium silicalite-1 (TS-1) zeolite with MFI topology is widely used for industrial applications due to the unique catalytic oxidation performance of the reaction system composed of TS-1 zeolite and H_2_O_2_ (TS-1/H_2_O_2_ system) [32]. The intrinsic activity of the Ti atom in the TS-1 is higher than that in the conventional titanium dioxide catalyst [33]. Zhen et al. synthesized a series of TS-1 zeolites with homogeneous distribution of Ti atoms and free of extra-framework Ti species and/or anatase, and these catalysts were used for the oxidation reactions. It was reported that there exists a molecular traffic control (MTC) effect in TS-1/H_2_O_2_ catalyst systems in catalytic oxidation reaction. The existence of a molecular traffic control (MTC) effect in TS-1/H_2_O_2_ resulted in lesser diffusion of the substrate molecules that would affect the catalytic oxidation behavior of TS-1 zeolite [34].

Optimizing the structural parameters like size, morphology and loading of metal nanoparticles on a solid support is of great significance because the solid support influences the catalytic performance, as it provides a large surface for dispersing metal species and also modifies the electronic and geometrical behavior of the metal nanoparticles via strong metal−support interactions [35,36]. Nanoparticles attract industrial interest in many distinct fields because of specific properties such as size, shape and distribution of nanoparticles, which influence the functional properties [37,38,39]. Magnetic nanoparticles (MNPs) with different shapes such as spheres, rods, tubes and flowers in porous and non-porous forms were synthesized [40,41,42]. Wang et al. studied the effect of localizing the metal nanoparticles within the zeolite and the shape selectivity to the metal nanoparticle catalysts and reported that the zeolite micropores could form a three-dimensional micropore modulation to the metal nanoparticles [43]. The unique properties of magnetite (Fe_3_O_4_) nanoparticles (Fe_3_O_4_ NPs), which include being super-paramagnetic, biodegradable and non-toxic to humans, draw great research interest [44,45,46]. Magnetic nanoparticles can be either used as single nanoparticles or made into composites by combining them with other materials. Magnetite zeolite composite can be made without any change in the magnetic properties because the modifications are not severed, and catalyst recovery is also feasible with simple techniques [47]. Loiola et al. have reported structural features of magnetic zeolites composites and discussed the importance of incorporation of magnetic nanoparticles for various applications [48].

The present study aimed to oxidize *p*-toluidine by hydrogen peroxide in the presence of magnetite supported on nanocrystalline titanium silicalite-1 (MNTS) zeolite catalysts at room temperature. The results are compared with magnetite supported nanocrystalline silicalite-1 (MNS) zeolite catalyst.

## 2. Results and Discussion

### 2.1. Dynamic Light Scattering (DLS) Measurements

The concentrated zeolite precursor sols (TCP-0 and SCP-0), aged concentrated zeolite precursor sols (NTS and NS), magnetite nanoparticles (M) and magnetite nanozeolite composite (M/NTS and M/NS) were evaluated by DLS analysis after dilution. The results presented in Table 1 represent that the particle size increases with the ageing period and the application of hydrothermal treatment, due to the aggregation of primary units [49].

### 2.2. X-ray Diffraction (XRD) Analysis

**Figure 1:**
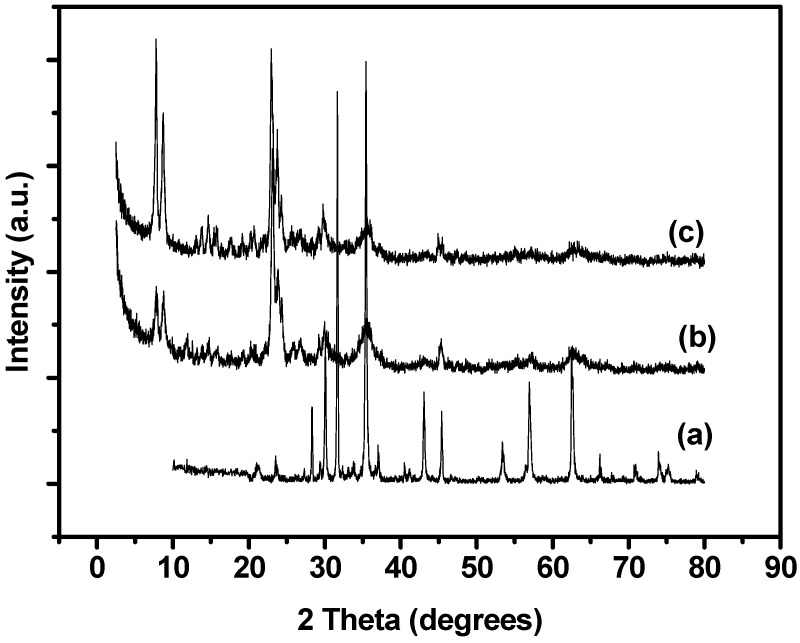
The XRD pattern: (a) magnetite nanoparticles, (b) M/NTS and (c) M/NS.

The XRD pattern (Figure 1a) of the synthesized magnetite nanoparticles reveals that the peaks located at 2θ values 30.31, 35.92, 43.6, 53.3, 57.38 and 62.96 correspond to (220), (311), (400), (422), (511) and (440) planes of the magnetite phase (JCPDS card #89-4319) [50]. It is important to note that there is no additional peak detected for other iron oxides, such as FeO and Fe_2_O_3_, which implies that the synthesized material is pure Fe_3_O_4_. In addition, the broad peak confirms the formation of nano-sized Fe_3_O_4_. Figure 1b,c show the XRD patterns of M/NTS and M/NS, which indicate that both samples are crystalline, and the reflection with high intensity observed in the 2θ ranges of 7–9°, 23–25° and 45° reveals the presence of magnetite and zeolite-MFI phases (JCPDS card #44-003) [51]. Also evident from the powder pattern is the highly crystalline nature of the formed zeolite–magnetite nanomaterial.

### 2.3. Scanning Electron Microscopy (SEM) Analysis

**Figure 2:**
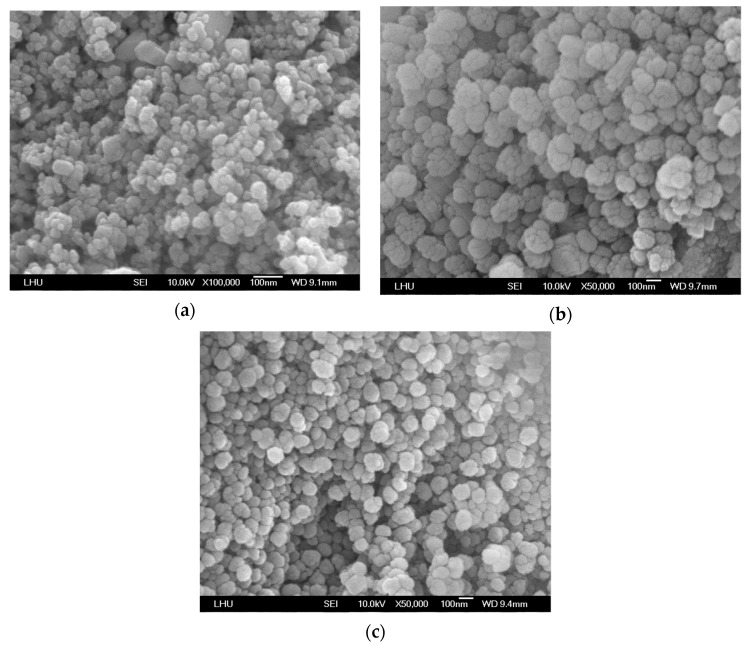
SEM micrographs: (**a**) magnetite nanoparticles, (**b**) M/NTS and (**c**) M/NS.

SEM analysis was used to confirm the morphology and textural properties of the synthesized materials. The SEM micrograph of magnetic nanoparticles is shown in Figure 2. The surface morphology of all the materials demonstrated the agglomeration of many ultrafine nanoparticles. Studying the surface morphology of magnetic nanoparticles shows that the iron particles have spherical morphology, as shown in Figure 2b, which confirmed the formation of the magnetite nanoparticles [52], and these particles are highly agglomerated, with diameter of about 30–50 nm (Figure 2a). Upon incorporation of magnetic nanoparticles into a zeolite network (M/NTS and M/NS), slight agglomeration can be observed, and this could be due to a large surface-area-to-volume ratio and interactions between magnetite and zeolite particles leading to morphological changes [53]. The well-dispersed particles have diameter of about 100 nm (Figure 2b,c).

### 2.4. Transmission Electron Microscopy (TEM) Analysis

**Figure 3:**
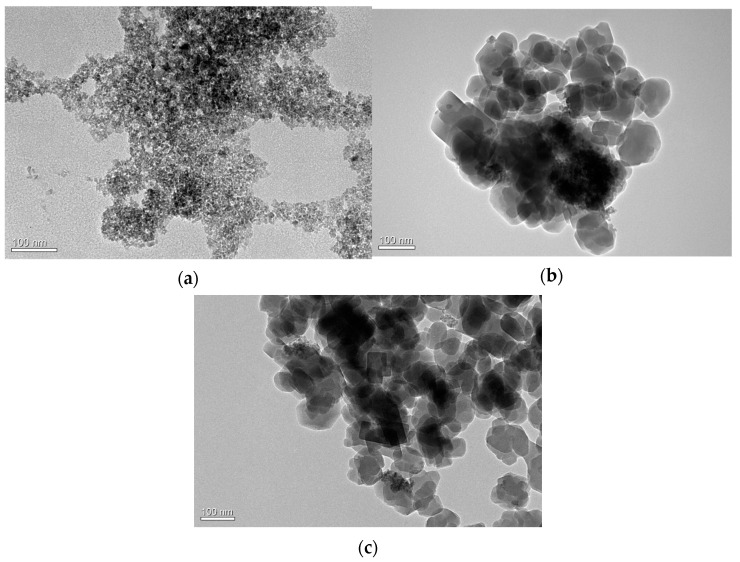
TEM micrographs: (**a**) magnetite nanoparticles, (**b**) M/NTS and (**c**) M/NS.

Figure 3 displays the TEM images of magnetite nanoparticles, M/NTS and M/NS materials. The magnetite sample exhibited spherical size with uniform particle size distribution in the range from 4 to 6 nm. The average size is determined as 5 nm (Figure 3a). Figure 3a,b show the TEM images of magnetite supported on titanium nanosilicate and magnetite supported on nanosilicate, respectively. It can be clearly seen that there is increase in particle size after coating with zeolite material. Large spherical contrast spots observed in the TEM image (Figure 3b,c) are considered to be the magnetite nanoparticles based on their particle size [54]. These magnetic nanoparticles are strongly attached to the zeolite surface and cannot be detached under high ultrasonication and at high temperature.

### 2.5. Surface Area and Pore Size Analysis

**Figure 4:**
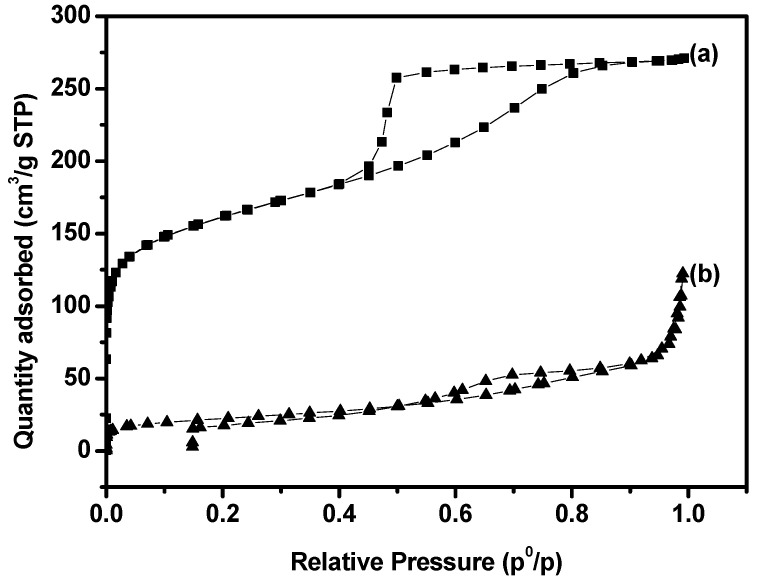
N_2_ adsorption–desorption isotherms of (a) M/NTS and (b) M/NS.

Nitrogen adsorption–desorption studies were carried out at 77.4 K to investigate the porous structure of the magnetite supported zeolite nanoparticles (M/NTS and M/NS). The nitrogen physisorption isotherms of M/NTS and M/NS nanoparticles are shown in Figure 4a,b. The results suggest that the samples are microporous [55]. The micropore volume is typically half the value for microporous ZSM-5. This implies that they are nanoparticles. The BET surface areas are found to be 512 and 568 m^2^/g for samples of M/NTS and M/NS. The nanozeolite materials are considered as highly porous materials with large surface area compared to other oxide nanoparticles [56]. The micropore volumes of M/NTS-1 and M/NS-1 are 0.127 cm^3^/g and 0.120 cm^3^/g, respectively.

### 2.6. Catalytic Activity Studies

**Scheme 1:**
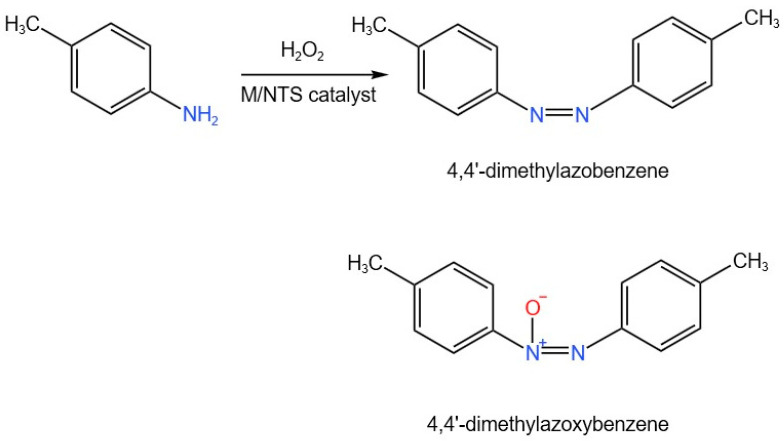
Oxidation of *p*-toluidine by hydrogen peroxide using M/NTS catalyst.

Oxidation of *p*-toluidine by hydrogen peroxide was carried out over the magnetite supported nanocrystalline zeolite (M/NTS and M/NS) catalysts. In the first step, the influence of time-on-stream (TOS) on the conversion was studied using the catalyst magnetite supported nanocrystalline titanium silicalite-1 (M/NTS-1) zeolite. The conversion of *p*-toluidine increased with increasing TOS until 100 min and became constant afterwards. Henceforth, for further study, the TOS was fixed at 100 min. The oxidation of *p*-toluidine in acetonitrile by hydrogen peroxide is carried out in the presence and absence of the M/NTS catalyst under identical conditions. The results indicate that without the catalyst, the reaction was extremely slow and produced only 4,4′-dimethylazoxybenzene. On the other hand, in the presence of the M/NTS catalyst, the reaction proceeded faster and produced 4,4′-dimethylazobenzene as a major product along with 4,4′-dimethylazoxybenzene. The reaction parameters such as *p*-toluidine/hydrogen peroxide molar ratio and catalyst quantity were optimized. Under the optimized reaction condition, the catalytic activity of magnetite supported nanocrystalline titanium silicalite-1 and magnetite supported nanocrystalline silicalite-1 were compared. The products, 4,4′-dimethylazobenzene (~85–95%) and 4,4′-dimethylazoxybenzene (~5–15%), were confirmed by gas chromatography–mass spectroscopy. The reaction scheme is represented as follows (Scheme 1). The formation of 4,4′-dimethylazobenzene and 4,4′-dimethylazoxybenzene compounds was also observed by several researchers in the oxidation of *p*-toluidine by hydrogen peroxide [57,58].

#### 2.6.1. Effect of Mole Ratio

**Figure 5:**
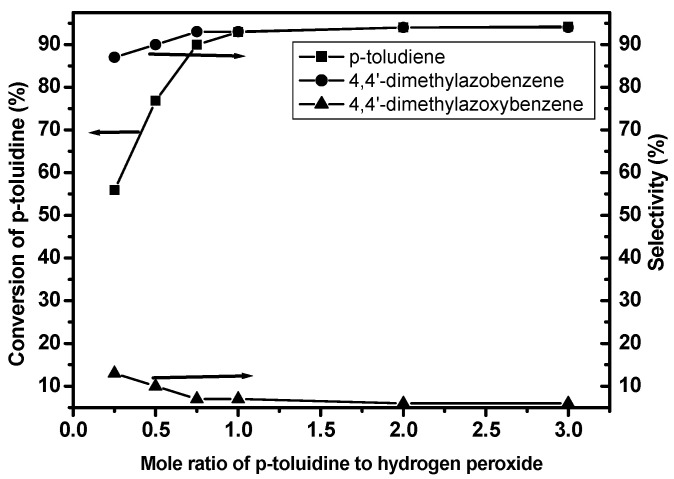
Effect of mole ratio of *p*-toluidine to hydrogen peroxide on the oxidation of *p*-toluidine by hydrogen peroxide over M/NTS catalysts. (Reaction condition: TOS, 100 min.; catalyst: M/NTS; catalyst weight: 0.15 g; reaction temperature: 25 °C).

The effect of the mole ratio of *p*-toluidine to hydrogen peroxide was studied from 0.25 to 3 by keeping the total volume constant. The results presented in Figure 5 illustrate that the % conversion of *p*-toluidine increased with increasing the mole ratio, reached a maximum of around 90% conversion at 0.75 mole ratio and then became econstant with further increasing the mole ratio. The amount of formation of products 4,4′-dimethylazobenzene and 4,4′-dimethylazoxybenzene varied at different molar ratios. The azo product increases from 87 to 94% and becomes constant beyond 0.75, and the azoxy product decreases 13% to 6% and becomes constant beyond 0.75 molar ratio. Henceforth, for further studies, the molar ratio of *p*-toluidine:hydrogen peroxide to be used was 0.75:1.

#### 2.6.2. Effect of Catalyst Quantity

**Figure 6:**
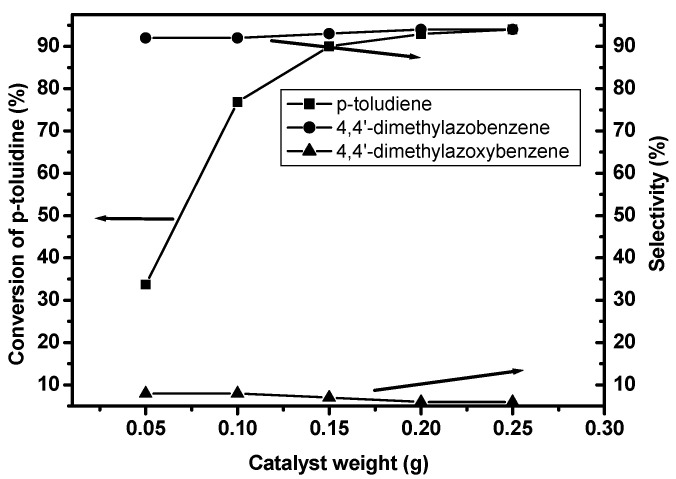
Effect of catalyst quantity on the oxidation of *p*-toluidine by hydrogen peroxide over M/NTS catalysts (reaction condition: TOS, 100 min.; catalyst: M/NTS; mole ratio of reactant: *p*-toluidine to hydrogen peroxide, 0.75:1; reaction temperature: 25 °C).

The amount of M/NTS catalyst needed for the catalytic oxidation of *p*-toluidine by H_2_O_2_ was optimized by studying the reaction at various amounts of catalysts in the range 0.05–0.25 g. Figure 6 presents the % conversion and % selectivity of products formed at various catalyst weights. The results illustrate that the % conversion raises from 33.7 to 93% when the catalyst quantity increases from 0.05 to 0.15 g and becomes constant at higher loadings. Regarding the product selectivity, the azo product increases from 92 to 94% and the azoxy product decreases from 8 to 6% when the catalyst quantity increases from 0.05 to 0.25 g. The increase in % conversion until 0.15 g catalyst weight represents the increase in active sites available for the reaction. The lesser influence of catalytic activity at higher catalyst loading signifies that additional active sites do not impact the catalytic reaction. The optimum catalyst weight is found to be 0.15 g, which was used for further studies.

#### 2.6.3. Reaction Mechanism

**Scheme 2:**
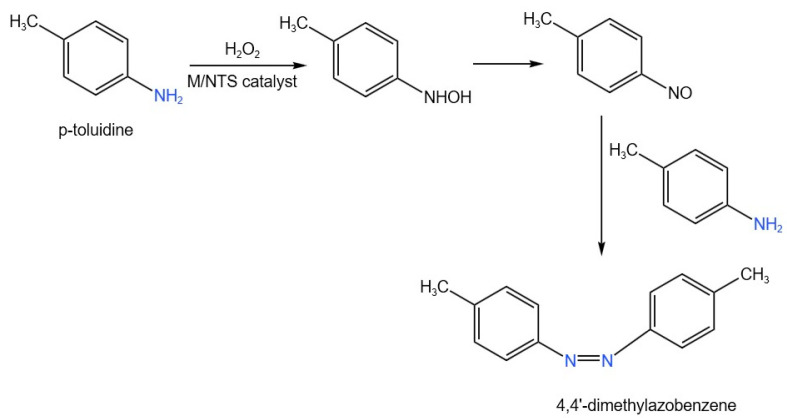
Mechanism of oxidation of *p*-toluidine by hydrogen peroxide with M/NTS catalyst.

The oxidation of *p*-toluidine by hydrogen peroxide in the presence of magnetite supported nanocrystalline titanium silicalite-1 (M/NTS) zeolite produces 4,4′-dimethylazobenzene and 4,4′-dimethylazoxybenzene. The 4,4′-dimethylazobenzene was formed through the nitroso compound, followed by coupling between the nitroso compound and the reactant, *p*-toluidine [57]. Scheme 2 illustrates the proposed reaction mechanism of oxidation of *p*-toluidine with H_2_O_2_ in the presence of M/NTS catalyst.

The oxidation of *p*-toluidine by hydrogen peroxide in the presence of M/NTS occurs rapidly, and 4,4′-dimethylazobenzene was formed in high yield compared to 4,4′-dimethylazoxybenzene, which shows that the coupling reaction between the nitroso compound and the *p*-toluidine occurs efficiently on the M/NTS catalyst. However, the oxidation of *p*-toluidine by hydrogen peroxide was extremely slow in the absence of M/NTS catalyst. This product is not formed in the absence of the catalyst. It is observed that the charge centers present in M/NTS catalyst accelerate the decomposition of hydrogen peroxide. The iron present in zeolite material is involved in the decomposition of hydrogen peroxide into hydroxyl radicals and hydroperoxyl radicals. In addition, the titanium silicates are used as catalyst for the oxidation of alkanes, alkenes and aromatic hydrocarbons with hydrogen peroxide under mild conditions [59]. The chemical structure of zeolite forms a network of channels and cavities, allowing easy penetration of molecules, so the reactants reach the active site and eliminate the products from the catalyst surface easily.

#### 2.6.4. Reusability of Catalyst

The magnetite supported nanocrystalline titanium silicalite-1 (M/NTS) zeolite catalyst’s reusability was studied three times, including the use of fresh catalyst. The used catalyst was centrifuged, washed with CH_2_Cl_2_ and subsequently dried at 120 °C for 6 h before being reused in subsequent batches. Table 2 presents the reusability of catalyst’s effect on conversion of *p*-toluidine and product selectivity. The results indicate that there is no appreciable change in the catalytic activity for up to three runs, including the fresh catalysts. It is concluded that the catalyst is stable and, hence, the catalyst is reusable.

#### 2.6.5. Comparison of Catalysts

Table 3 compares the performance of magnetite supported nanocrystalline titanium silicalite-1 (M/NTS) zeolite catalyst and magnetite supported nanocrystalline silicalite-1 (M/NS) zeolite catalysts for the oxidation of *p*-tolidine. In order to compare the catalytic activity of M/NTS and M/NS, a set of reactions were carried out at identical conditions of 0.15 g catalyst, 0.17:1 *p*-toluidine:H_2_O_2_ molar ratio and reaction temperature at 25 °C. The results are presented in Table 3. The results indicate that M/NTS showed higher conversion compared to M/NS catalysts due to the presence of titanium in the framework of zeolite NTS-1.

The catalytic activity of oxidation of *p*-toluidine was compared with previously reported results. In the past, few studies have been reported using conventional homogeneous catalysts such as KMnO_4_, K_3_Fe(CN)_6_, K_2_Cr_2_O_7_, K_3_Fe(CN)_6_, enzyme peroxidase, dilute sulfuric acid and FeCl_3_, and these catalysts produced trimers and tetramers [10,11,12,13,14]. In a recent study, it was reported that the oxidation of *p*-toluidine in acetonitrile by hydrogen peroxide in the presence of functionalized multiwalled carbon nanotubes (CNT) produced 4,4′-dimethylazobenzene and 4,4′-dimethylazoxybenzene. With functionalized multiwalled carbon nanotubes catalyst, the azo compound was much higher than the azoxy compound, viz. 2.5% azo and 4.2% azoxy of % product ratio [57]. But without the catalyst, only azoxy compound was reported. These results are consistent with the present study on M/NTS and M/NS catalysts. However, the ratio of azo and azoxy products is much higher in M/NTS and M/NS catalysts compared to previously reported catalyst on functionalized multiwalled carbon nanotubes (Table 3). The higher activity with high product selectivity of azo compound with zeolite-based catalysts is due to faster adsorption of reactants and desorption of products on porous nanocrystalline zeolite materials.

## 3. Materials and Methods

### 3.1. Chemicals

Tetraethoxysilane (TEOS), tetrabutylorthotitanate (TBOT), tetrapropylammonium hydroxide (TPAOH, 40%aq), ferric chloride (FeCl_3_·6H_2_O), sodium carbonate, ascorbic acid, *p*-toluidine, acetonitrile and hydrogen peroxide 30% were commercial samples from Merck and were used without further purification.

### 3.2. Synthesis of Magnetite Nanoparticles

The hydrophilic magnetite nanoparticles were prepared from hydrated ferric chloride (FeCl_3_.6H_2_O) by dissolving 0.55 g in 25 mL H_2_O with continuous stirring [60]. Then, 10 mL of Na_2_CO_3_(0.6M) was added drop by drop to the ferric chloride solution with continuous stirring for 10 min. To the above solution, ascorbic acid (12 g) was added while stirring. The autoclave was kept at 160 °C for 3 h and then allowed to cool in air naturally. The solid was rinsed thoroughly by three subsequent steps: washing in deionized water/washing in alcohol/centrifugation. The final products were separated from the reaction medium by centrifugation and dried overnight at 60 °C in an oven. The dried magnetite sample is denoted as M.

### 3.3. Synthesis of Nanocrytalline TS-1

To prepare nanocrystalline TS-1, a clear solution of TPAOH-TiO_2_-SiO_2_-H_2_O was prepared at room temperature as described in the literature [55]. In typical procedure, 10.4 g Tetraethylorthosilicate and 12.62 g tetrabutylorthotitanate were taken in a polypropylene bottle and stirred at 25 °C until a homogeneous mixture was formed. This solution is denoted as solution A. About 6.4 g tetrapropylammonium hydoxide (TPAOH) (40 wt%) was taken in a beaker and dissolved in 8.5 g water. This solution is denoted as solution B. Solution B was added slowly into solution A at a rate of 1.5 mL/min while stirring continuously for about 2 h in order to obtain a clear solution. To this solution mixture, 8.5 g of water was added, so the final molar composition obtained was 0.25TPAOH:0.06TiO_2_:1.00SiO_2_:20H_2_O, and the pH of this solution was 11.8. This solution was concentrated using rotary evaporator at 80 °C. The concentrated precursor sol (TCP-0) was kept aside at RT, and the rest was poured into PP vial and aged at 80 °C for 30 h. The product of aged treatment was translucent and denoted as NTS.

### 3.4. Synthesis of Nanocrytalline Silicalite-1

The clear sol of silicalite-1 (3 g oxide) was prepared as follows: 10.4 g of TEOS (tetraethoxysilane, Merck, Mumbai, India) was added to a 250 mL PP (polypropylene) bottle containing 6.34 g of TPAOH (40% aq. Merck), followed by the addition of 16 g of DI water, and the solution was stirred in a magnetic stirrer for about 2 h to hydrolyze TEOS so that a clear solution was formed. The molar composition of the resulting sol was 1 SiO_2_/4 EtOH/0.25 TPAOH/15 H_2_O. The pH of the final solution was 11.7. The clear sol was concentrated to transparent viscous sol (SCP-0) in a rotary evaporator at 80 °C for 50 min. The mole ratio of water to silica after concentration was 4.01. The pH of the final concentrated NPs solution was 12.9. The concentrated transparent viscous sol was heated at low temperature (80 °C) in an air oven for 6 h. The product of aged treatment was translucent and denoted as NS.

### 3.5. Synthesis of Magnetite Supported Nanocrystalline Zeolite

Magnetite (Fe_3_O_4_) nanoparticles were dispersed in water (10 wt%) and added to the aged zeolite precursor solutions individually (MNTS and MNS) in the molar ratio of 1:1 under stirring. The mixture was hydrothermally treated at 175 °C for 30 min. The product was collected using a magnet. The samples were calcined in air at 550 °C (5 h, 2 °C/min) and coded as M/NTS and M/NS. The elemental compositions of the M/NTS sample include O (70.91%), Ti (0.55%), Si (5.35%) and Fe (6.58%) as determined by ICP-AES analysis.

### 3.6. Characterization Methods

X-ray Diffraction (XRD) analysis was performed to determine the crystallinity and identity of zeolite MFI structure and iron oxide phases. XRD patterns were obtained with a Rigaku 2000 diffractometer (Tokyo, Japan) using Cu-Ka radiation with a wavelength of 1.5418 A°. The scans were taken from 2.5 to 80° at a scanning speed of 2° deg/min. The particle size distribution and Zeta potential were analyzed by dynamic light scattering (DLS, ZetaSizer-3000 with a 10 Mw He-Ne Laser from Malvern Instrument Co., (Malvern Instruments, Malvern, UK)). Morphology and particle size examinations of the samples were carried out using field emission scanning microscope (FESEM) JEOL SM-6500f and TEM (JEM-2010, 200 kV). Nitrogen adsorption measurements were carried out at 77.4 K on a Micromeritics ASAP 2010 instrument (Norcross, GA, USA). The elemental composition of the M/NTS material was obtained using inductively coupled plasma–atomic emission spectroscopy (ICP-AES).

### 3.7. Catalytic Studies

Oxidation of *p*-toluidine by hydrogen peroxide in the presence of magnetite supported nanaocrystalline titanium silicalite-1 (M/NTS) zeolite catalysts was carried out in a magnetically stirred glass reactor (capacity 100 cm^3^) fitted with a thermometer for measuring the reaction temperature. The reactor was kept in a constant-temperature water bath. In a typical experiment, the reaction mixture consisted of 0.75 M *p*-toluidine solution in acetonitrile and equal concentration of hydrogen peroxide solution, which were taken in a round-bottomed flask. Freshly activated catalyst (0.15 g) was added, and the flask with its contents was kept at ambient temperature (25 °C) in a water bath for 100 min and stirred magnetically. The progress of the reaction was monitored by withdrawing samples from the reactor and analyzed by gas chromatographic analysis (Hewlett-Packard 5890), using a DB-1 column and a FID detector. The products, 4,4′-dimethylazobenzene and 4,4′-dimethylazoxybenzene, were detected by gas chromatography–mass spectroscopy (GC-MS).

## 4. Conclusions

The catalytic activity of magnetite supported nanocrystalline titanium silicalite-1 (M/NTS) zeolite and magnetite supported nanocrystalline silicalite-1 (M/NS) catalysts for the liquid phase oxidation of *p*-toluidine by hydrogen peroxide was studied at ambient temperature. The environmental hazards resulting from the conventional manufacturing procedures using classical catalysis have forced us to seek less polluting catalytic processes for the oxidation of *p*-toluidine by hydrogen peroxide for the production of 4,4′-dimethylazobenzene. The oxidation of *p*-toluidine by hydrogen peroxide without catalyst is a very slow reaction that produces only 4,4′-dimethylazoxybenzene, whereas magnetite supported on nanocrystalline titanium silicalite-1 (M/NTS) zeolite catalyst under identical conditions produces 4,4′-dimethylazobenzene as major product (~85–95%) and 4,4′-dimethylazoxybenzene as minor product (~5–15%). Optimizing the reaction parameters indicates that the nanocrystalline titanium silicalite-1 (M/NTS) zeolite catalyst plays a significant role because, in addition to large surface for dispersing metal species, it also modifies the electronic and geometrical behavior of the metal nanoparticles via strong metal−support interactions [27,28]. Comparison of M/NTS and M/NS catalysts for the oxidation of *p*-toluidine by hydrogen peroxide showed that the M/NTS catalyst displayed higher activity than the M/NS catalyst. The higher activity with high product selectivity of azo compound was due to faster adsorption of reactants and desorption of products on porous nanocrystalline titanium silicalite-1 materials.

## Figures and Tables

**Table 1:** Dynamic Light Scattering (DLS) Measurements.

Sample	Particle Size (nm)	Polyindex
M	4.1	0.02
TCP-0	4.2	0.58
SCP-0	4	0.54
NTS	39	0.05
NS	36.9	0.03
M/NTS	46.7	0.04
M/NS	43.6	0.03

**Table 2:** Effect of reusability of the catalyst on conversion of *p*-toluidine and product distribution.

Catalyst (M/NTS)	Conversion of *p*-Toluidine	Product Distribution	
		4,4′-Dimethylazobenzene	4,4′-Dimethylazoxybenzene
Fresh catalyst	90	93	7
1st reuse	88.7	93	7
2nd reuse	88	93	7

(Reaction condition: TOS, 100 min.; catalyst: 0.15 g of M/NTS; mole ratio of reactant: *p*-toluidine to hydrogen peroxide, 0.75:1; reaction temperature: 25 °C).

**Table 3:** Comparison of catalytic activity of M/NTS and M/NS zeolite catalysts with oxidation of *p*-toluidine.

Catalyst	Conversion of *p*-Toluidine	Product Distribution	
		4,4′-Dimethylazobenzene	4,4′-Dimethylazoxybenzene
M/NTS	90	93	7
M/NS	67.8	88	12

(Reaction condition: TOS, 100 min.; catalyst: 0.15 g of M/NTS; mole ratio of reactant: *p*-toluidine to hydrogen peroxide, 0.75:1; reaction temperature: 25 °C).

## Data Availability

The data presented in this study are available on request from the corresponding author.

## Sample Availability

Samples of the compounds are available from the authors.
